# Plasma phospholipid fatty acid profile confirms compliance to a novel saturated fat-reduced, monounsaturated fat-enriched dairy product intervention in adults at moderate cardiovascular risk: a randomized controlled trial

**DOI:** 10.1186/s12937-017-0249-2

**Published:** 2017-05-23

**Authors:** Oonagh Markey, Dafni Vasilopoulou, Kirsty E. Kliem, Albert Koulman, Colette C. Fagan, Keith Summerhill, Laura Y. Wang, Alistair S. Grandison, David J. Humphries, Susan Todd, Kim G. Jackson, David I. Givens, Julie A. Lovegrove

**Affiliations:** 1 Hugh Sinclair Unit of Human Nutrition and Institute for Cardiovascular and Metabolic Research, University of Reading, Reading, RG6 6AP UK; 2 Department of Food and Nutritional Sciences, University of Reading, Reading, RG6 6AP UK; 3 Animal, Dairy and Food Chain Sciences, University of Reading, Reading, RG6 6AP UK; 4 MRC Elsie Widdowson Laboratory, 120 Fulbourn Road, Cambridge, CB1 9NL UK; 5 NIHR BRC Nutritional Biomarker Laboratory, University of Cambridge, Cambridge, CB2 0QQ UK; 6 Department of Mathematics and Statistics, School of Mathematical, Physical and Computational Sciences, University of Reading, Reading, RG6 6AX UK; 7 Institute for Food, Nutrition and Health, University of Reading, Reading, RG6 6AR UK; 8 Present address: School of Sport, Exercise and Health Sciences, Loughborough University, Loughborough, LE11 3TU UK

**Keywords:** Cardiovascular disease, Dairy products, Dietary fat composition, Food-exchange model, Fatty acids, Monounsaturated fatty acids, Nutrition assessment, Phospholipids, Saturated fatty acids

## Abstract

**Background:**

Dairy products are a major contributor to dietary SFA. Partial replacement of milk SFA with unsaturated fatty acids (FAs) is possible through oleic-acid rich supplementation of the dairy cow diet. To assess adherence to the intervention of SFA-reduced, MUFA-enriched dairy product consumption in the RESET (**RE**placement of **S**aturat**E**d fat in dairy on **T**otal cholesterol) study using 4-d weighed dietary records, in addition to plasma phospholipid FA (PL-FA) status.

**Methods:**

In a randomised, controlled, crossover design, free-living UK participants identified as moderate risk for CVD (*n* = 54) were required to replace habitually consumed dairy foods (milk, cheese and butter), with study products with a FA profile typical of retail products (control) or SFA-reduced, MUFA-enriched profile (modified), for two 12-week periods, separated by an 8-week washout period. A flexible food-exchange model was used to implement each isoenergetic high-fat, high-dairy diet (38% of total energy intake (%TE) total fat): control (dietary target: 19%TE SFA; 11%TE MUFA) and modified (16%TE SFA; 14%TE MUFA).

**Results:**

Following the modified diet, there was a smaller increase in SFA (17.2%TE vs. 19.1%TE; *p* < 0.001) and greater increase in MUFA intake (15.4%TE vs. 11.8%TE; *p* < 0.0001) when compared with the control. PL-FA analysis revealed lower total SFAs (*p* = 0.006), higher total *cis*-MUFAs and *trans*-MUFAs (both *p* < 0.0001) following the modified diet.

**Conclusion:**

The food-exchange model was successfully used to achieve RESET dietary targets by partial replacement of SFAs with MUFAs in dairy products, a finding reflected in the PL-FA profile and indicative of objective dietary compliance.

**Trial registration:**

ClinicalTrials.gov Identifier: NCT02089035, date 05-01-2014.

**Electronic supplementary material:**

The online version of this article (doi:10.1186/s12937-017-0249-2) contains supplementary material, which is available to authorized users.

## Background

Cardiovascular diseases (CVD) are one of the leading causes of mortality in the UK [1]. As a result of the clear link between a high intake of SFAs and elevated LDL-cholesterol concentrations, dietary guidelines for CVD prevention advocate reducing SFA intake to ≤10% of total energy (%TE) [2, 3]. Despite recommendations, the UK adult population still exceeds the target for dietary SFA intake, with a mean intake of 12.1%TE [4].

Dairy products are major sources of dietary SFA and account for up to 35% of total UK SFA intake [4] and therefore reducing consumption of regular-fat dairy foods or replacing them with lower fat or fat-free alternatives is often advised [5, 6]. However, prospective studies have not presented consistent evidence for an adverse link between higher consumption of milk and dairy products and increased risk of CVD, regardless of milk fat content [7–10]. Furthermore, this rationalisation does not acknowledge the complex nature of the dairy food matrix, which may be fundamental to cardiovascular health [11]. Dairy fat contains a complex mixture of fatty acids (FA) including SFAs, MUFAs, PUFAs, *trans*-fatty acids (TFAs) and branched-chain FAs [12, 13]. Furthermore, milk is rich in micronutrients and bioactive peptides, which have been reported to exert cardio-protective effects [14, 15].

While the findings of some recent epidemiological studies have challenged the traditional link between SFA and coronary heart disease risk, and mortality [16–18], it is important to consider the macronutrient that replaces energy from dietary SFA [18, 19]. There is evidence from a randomized controlled trial (RCT) that replacement of SFA with PUFA had minimal but increased effects on CHD mortality [20]. However a more recent systematic review and meta-analysis of RCT suggests that lowering SFA intake could reduce cardiovascular events by 17%, with SFA replacement with PUFA estimated to reduce these events by 27% [21]. Despite these apparent discrepancies, current US and EU dietary guidelines recommend reduction of SFA and replacement with PUFA and/or MUFA respectively [22, 23]. In support of the latter review and dietary recommendations, a RCT illustrated that replacing SFA with *n*-6 PUFA for 16-weeks induced a reduction in serum concentration of LDL-cholesterol (a major risk factor for CVD) by 13.6%, with a comparable reduction of 11.3% on replacement of SFA with MUFA [19].

It is well documented that partial replacement of milk SFA with unsaturated FAs, predominantly in the form of MUFA, through supplementation of the dairy cow diet with plant oils or oil seeds is feasible [24–26]. At a population level, this initiative could provide a sustainable means of reducing SFA in dairy products, whilst limiting the entry of SFA into the wider food chain. While limited evidence from human studies suggest that consumption of dairy products with a modified FA profile may beneficially impact on CVD risk, there has been a heavy reliance on fasting lipid biomarkers as sole predictors of risk [27]. Further research is needed to elucidate whether FA-modified dairy product consumption has a differential effect on more novel risk markers, including endothelial function, arterial stiffness, systemic inflammation and ambulatory blood pressure, when compared with dairy products of typical milk FA composition [15, 27].

The REplacement of SaturatEd fat in dairy on Total cholesterol (RESET) study was conducted to investigate the chronic and acute effects of two iso-energetic high-fat, high-dairy diets, which varied in FA composition, on traditional and novel cardiometabolic risk markers in free-living individuals. It is important to evaluate strategies employed for the achievement of dietary targets in controlled human intervention studies [28]. The purpose of the current paper is to describe the RESET dietary exchange strategy that was developed to enable manipulation of the FA profile of the diet over two 12-week periods, through the use of SFA-reduced, MUFA-enriched (modified) dairy products and matched control dairy foods with a FA profile typical of retail products. Although it is recognised that specific plasma FA levels can be indicative of dietary FA consumption, the composition of the plasma phospholipid FA (PL-FA) fraction is believed to be a good biomarker of FA intake over recent days to weeks and an objective indicator of dietary compliance [29–31]. Thus, this paper aims to report the chronic dietary intervention food-exchange model and the compliance to the FA-modified and control dietary exchange periods using 4-d weighed dietary records, self-reported daily tick-sheets and PL-FA.

## Materials and methods

### Study participants and design

The RESET study was a double-blinded, randomised, controlled, crossover designed trial registered at Clinicaltrials.Gov as: NCT02089035. The study was given a favourable ethical opinion for conduct by the University of Reading’s Research Ethics Committee (13/43) and was conducted according to the guidelines of the Declaration of Helsinki. All participants provided written informed consent prior to study entry.

Men and women aged 25–70 years were recruited from the Berkshire area of the UK in three cohorts between February 2014 and April 2016. A modified Framingham risk prediction algorithm was employed to identify individuals with moderate CVD risk [31, 32]. To meet inclusion criteria, participants were required to have ≥ 2 CVD risk points, a score that suggested a 50% greater risk of CVD development than the population mean [32]. Briefly, this score was calculated based on the existence of single or multiple CVD risk factors, including elevated fasting total cholesterol, glucose, systolic/diastolic blood pressure, low HDL-cholesterol, overweight/obesity or a family history of myocardial infarction. Potential participants were also required to meet the following inclusion criteria: BMI 19–32 kg/m^2^; blood pressure <160/100 mmHg; total cholesterol <8 mmol/L; haemoglobin: >125 g/L for women and 135 g/L for men; normal liver and kidney function; not pregnant or lactating; no dietary supplementation; no lactose or dairy intolerances/allergies; not taking medication for hyperlipidaemia, hypertension, hypercoagulation or inflammatory conditions; no diagnosis of myocardial infarction, stroke or diabetes; participating in <20 min × 3 times/week of vigorous aerobic activity and not consuming excessive amounts of alcohol (men: <21 units/week; women: <14 units/week). If known not to interfere with study outcomes, participants continued to take their regular prescribed medication, without changes in dosage, for the duration of the study.

### Study foods

The FA composition of the dairy products (including ruminant *cis* and TFAs), along with the methods for their production, will be described in detail elsewhere (Kliem KE, Humphries DJ, Markey O, Vasilopoulou D, Fagan CC, Grandison AS, Todd S, Givens DI, Lovegrove JA: Food chain approach to lowering saturated fat in milk and dairy products: the RESET study, submitted). Based on a similar feeding strategy, it was estimated that our bovine intervention would increase *cis*-MUFA in the milk from 20 to 30 g/100 g FA, while reducing SFA from 70 to 55–60 g/100 g FA [33]. Briefly, the diet of recruited Holstein-Friesian cows was supplemented with approximately 1 kg high-oleic sunflower oil (AAK Ltd., Hull, East Yorkshire, UK) per cow per day for a ≥ 28-d period to produce milk which had a portion of SFA replaced with MUFA. Subsequently, raw milk was used to produce SFA-reduced, MUFA-enriched (modified) ultra high temperature (UHT) milk, Cheddar cheese and butter. Raw milk, provided by Arla UK Plc (Taw Valley Creamery, North Tawton, UK), was used to produce control UHT milk. Control Cheddar cheese and butter, with a FA profile typical of retail products, were also supplied by Arla UK Plc (Taw Valley Creamery, North Tawton, UK).

### Dietary intervention

The technique of minimization, controlling for gender, age, BMI and total cholesterol was used to randomly allocate participants to one of two groups in the study, with Group 1 being assigned to receive Diet A (Modified) and then Diet B (Control) during their first and second dietary intervention periods, respectively and vice-versa for Group 2 [34]. In each group, participants completed two 12-week dietary intervention periods, separated by an 8-week washout period. One dietary intervention was an iso-energetic high-fat daily dietary exchange (dietary target: 38%TE total fat) which was achieved by replacing habitual dairy foods, cooking oil and snacks with SFA-reduced, MUFA-enriched UHT milk, Cheddar cheese and butter (modified). The second dietary intervention used matched products with a FA profile typical of retail products (control). The dietary exchange periods were rich in dairy foods and were designed to give diets of equal fat content that varied in SFA and MUFA composition. The dietary target intake for total fat was 38%TE in both diets, with specific dietary FA targets for the control (19%TE SFA and 11%TE MUFA) and modified diets (16%TE SFA and 14%TE MUFA).

Measurements of circulating total cholesterol, composed of LDL and HDL-cholesterol (primary outcome), and other established and novel CVD and cardiometabolic risk markers were assessed prior to and after each dietary intervention period (chronic study). This manuscript will present the 4-d weighed dietary records, tick-sheet records, anthropometric measures, and PL-FA analysis to assess dietary compliance. The other clinical outcome measures from the RESET study will be published elsewhere.

### Food-exchange model

The RESET study food-exchange strategy for reducing SFA intake was designed based on a model adapted from both the Dietary Intervention and Vascular Function (DIVAS) study and the Reading, Imperial, Surrey, Cambridge, and Kings (RISCK) study [31, 35], which were based on the National Diet and Nutrition Survey (NDNS) for adults (aged 19–64 years) [36]. The mean habitual energy, total fat, SFA, MUFA and PUFA intakes of participants from the RISCK study were used [35], with additional TFA data obtained from the DIVAS study [31], as these dietary data represented the intake of UK adults who were at increased or moderate CVD risk. Added oils, added fats (butter and spreads), milk, cheese as well as sweet and savoury snacks were identified as ‘exchangeable dietary fat’ sources which could be removed from the diet and replaced with study foods. The total contribution of these ‘exchangeable fat’ food groups to mean daily energy, fat and FA intake were estimated, based on mean population data from the 2000/2001 NDNS ‘percentage contribution of types of foods’ (Table 1). The total exchangeable fat was subtracted from the RISCK/DIVAS study habitual energy, total fat and FA intake to calculate non-modifiable fat intake. This was employed to form the backbone of the food-exchange model, onto which the RESET study foods could be added to create two iso-energetic dietary exchange periods of dairy products that varied in FA composition (Table 2).

**Table 1:** The RESET food-exchange model^a^

	Total energy	Total fat	SFA	MUFA	TFA	PUFA
	(MJ/d)					
Total habitual intake (including alcohol)^b^, g/d	8.25	80.1	29.6	26.6	1.0	13.1
Total habitual intake, %TE		36.6	13.5	12.1	0.4	6.0
Exchangeable fat intake						
Added oils, g/d	0.35	8.5	0.8	3.3		4.0
Added fats (butter and spreads), g/d	0.35	8.6	3.3	2.9	0.2	1.7
Milk, g/d	0.45	4.0	2.4	1.0	0.0	0.3
Cheese, g/d	0.25	4.5	2.8	1.0	0.1	0.1
Sweet and savoury snacks, g/d^c^	0.87	10.0	4.4	3.4	0.1	0.6
Total exchangeable fat intake, g/d	2.27	35.5	13.6	11.6	0.4	6.6
Total exchangeable fat intake adjusted for habitual intake, g/d	2.22	38.5	14.4	12.5	0.4	7.3
Non-exchangeable fat intake, g/d	6.03	41.7	15.3	14.1	0.6	5.9

*TFA trans*-fatty acids, *%TE* percentage of total energy

^a^Adapted from Weech et al. [31]

^b^Mean daily dietary intakes (total energy, total fat, SFA, MUFA and PUFA) from a population with increased cardiovascular disease risk [35]. Mean daily dietary TFA intake from a population at moderate cardiovascular disease risk [31]

^c^Included buns, cakes, pastries, potato chips and chocolate confectionary

**Table 2:** Replacement model for diets containing control and modified dairy products for use in the RESET study^a^

	Quantity	Total energy	Total fat	SFA	MUFA	TFA^b^	PUFA
	(g/d)	(MJ/d)					
Non-exchangeable fat intake, g/d		6.03	41.7	15.3	14.1	0.5	5.9
Control dietary exchange							
Exchangeable fat intakes^c^							
Butter, g/d	21.5	0.65	17.4	11.1	4.5	0.7	0.6
Cheese, g/d	45.0	0.76	15.1	10.2	3.5	0.5	0.4
Milk, g/d	340.0	0.75	8.6	5.7	2.1	0.3	0.3
Total intake, g/d		8.19	82.8	42.3	24.2	2.0	7.2
Total intake, %TE			38.1	19.4	11.1	0.9	3.3
Target intake, %TE			38.0	19.0	11.0	0.9	
Modified dietary exchange							
Exchangeable fat intakes							
Butter	25.1	0.76	20.4	10.2	8.1	2.1	0.7
Cheese, g/d	45.0	0.69	12.6	6.3	5.0	1.4	0.4
Milk, g/d	340	0.79	8.8	4.4	3.6	0.9	0.3
Total intake, g/d		8.27	83.4	36.0	30.8	4.9	7.3
Total intake, %TE			38.0	16.4	14.0	2.1	3.3
Target intake, %TE			38.0	16.0	14.0	2.0	
Mean difference between dietary exchange periods, %TE			−0.6	6.2	−6.7	−2.9	−0.2

The FA composition of dairy products was determined using a GC-flame ionisation detection method [38], with a conversion factor of 0.933 used to estimate the proportion of FAs in the total fat content of each product [39]

%TE, percentage of total energy

^a^Total intake is the sum of exchangeable and non-exchangeable intakes based on MJ/d for energy and grams/d for FAs

^b^Total TFA intake (%TE) is calculated based on recorded energy intake at baseline (MJ/d) in Weech et al. [31]

^c^Energy and fat content of the dairy products is based on nutritional analysis conducted by SGS United Kingdom Ltd. (Ellesmere Port, Cheshire)

### Implementation of intervention diets

After completing baseline visits (i.e. at the beginning of each intervention period), participants were provided with 1:1 dietary advice on how to replace dietary fat sources in their habitual diets (e.g. added oils, milk, cheese, sweet and savoury snacks) with the study dairy foods. They were also given dietary guidelines and recipe suggestions to take home. Care was taken to ensure that no study visits were arranged during or immediately after the Christmas period (mid-December to mid-January). Where it was not feasible to avoid holidays and business trips during intervention periods, participants were given instructions on how to travel with the study products. This was made more convenient by providing UHT milk that did not require refrigeration. Furthermore, participants were provided with frozen butter and were advised that they could it frozen during transit, with the use of ice packs. Products were provided in plain packaging and were only identifiable by a code (A or B), to ensure that participants and researchers remained blinded to the intervention arms.

At the beginning of each intervention period, participants were given adequate study dairy foods for a 4-week period. They attended the Hugh Sinclair Unit of Human Nutrition for a food collection visit at weeks 4 and 8. During this visit, adherence to the dietary intervention was assessed by reviewing completed tick-sheet records and any issues were discussed and resolved. In addition, investigators recorded the participant’s weight and any changes ≥ ± 1 kg of baseline, were addressed through advice to alter snack, meat and/or carbohydrate intake. Participants were asked to maintain their habitual physical activity levels during each intervention period. At the end of the first intervention period, participants were asked to return unopened, leftover study products and were asked not to consume any leftover study products for the duration of the washout period.

#### Assessment of dietary intake

Participants received verbal and written instructions by the investigators for recording 4-d weighed dietary records approximately 2 week before the first study visit (week 0). Investigators also provided participants with examples of completed diaries, including how to record recipes, and digital scales for recording food intake. Exceptions for weighing included food consumed outside of the home. On these occasions, portion-size images were used to estimate consumed portions and subsequently quantified using published food portion tables [37]. Participants completed diet diaries on four separate occasions (weeks 0, 11, 19 and 31): habitual diet intake was represented from baseline diet diaries (weeks 0 and 19), while diaries completed during weeks 11 and 31 represented participant compliance to the intervention diets. Prior to visit 1, food intake was recorded on three weekdays and one weekend day. The same days were repeated for subsequent diaries. Assessment of completion of the diaries was undertaken by the investigators during study visits. If necessary, additional information was requested to facilitate precise data entry.

Food diaries were analysed using the NDS Nutrient Database or McCance and Widdowson’s (MW7) nutrient databank contained within the nutrient analysis software Dietplan 7 (Forestfield Software Ltd.). The nutritional content of the control and modified dairy products is presented in Kliem et al. (Kliem KE, Humphries DJ, Markey O, Vasilopoulou D, Fagan CC, Grandison AS, Todd S, Givens DI, Lovegrove JA: Food chain approach to lowering saturated fat in milk and dairy products: the RESET study, submitted). Energy and macronutrient content (Group 1 nutritional analysis) was performed in duplicate by SGS UK Ltd. (Ealing, London, UK; ISO 17025 accredited laboratory). Analysis of sodium, calcium, magnesium and phosphorus content was conducted in duplicate by inductively coupled plasma-optical emission spectrometry at Quaternary Scientific (QUEST, School of Archaeology, Geography and Environmental Science, University of Reading, Reading, UK). Extracted lipids from milk, cheese and butter samples were analysed in triplicate for FA composition using a GC-flame ionisation detection method [38]. A conversion factor of 0.933 was used to estimate the proportion of FAs in the total fat content of each dairy product [39]. Subsequently, the quantity of SFA, MUFA, TFA and PUFA were calculated per daily portion of each study dairy product (g/d). Nutrient composition of the study products was entered manually in Dietplan. For the purpose of analysis, the mean daily intakes of energy and macronutrients were recorded and the %TE was calculated to adjust for energy intake (EI). Dietary fibre intake was defined using the Association of Official Analytic Chemists (AOAC) method [40].

### Assessment of underestimation of energy intake

Determination of possible underestimation of dietary EI was assessed for each participant. Basal metabolic rate, based on age, gender and body weight, was estimated using the Henry equation [41]. A sedentary lifestyle was represented by a physical activity level score of 1.2 [31, 42]. The Goldberg lower 95% confidence limit was calculated as <1.132 using the CV recommended by Black (*n* = 51; 4 d) and was used to identify under-reporters of EI [42].

### Assessment of dietary compliance

As outlined in Table 2, participants were required to consume the minimum daily portions of each of the following study products: 340 g/d milk, 45 g/d cheese and 21.5 or 25.1 g/d of control or modified butter, respectively. This ensured that the intervention diets were iso-energetic and contained equal quantities of dairy fat (38%TE total fat; approx. 41 g/d). As a means of monitoring compliance with each study product type, participants were required to complete tick-sheet records on a daily basis throughout each 12-week dietary intervention period. Participants were given the option of marking the tick box if they had consumed the required portion size or could choose to record the actual weight of the product consumed. In order to calculate dietary compliance, one point was subtracted for each day that participants had not consumed a study product. These points were summed over each intervention period and were used to calculate percentage dietary compliance for each product type.

### Assessment of anthropometrics

Participants were requested to fast overnight for 12 h before each clinical visit, following consumption of a standardised low-fat evening meal (<1.46 MJ; < 7 g total fat) and low-nitrate water (Buxton Mineral Water, Nestlé Waters, Buxton, UK). At weeks 0, 12, 20 and 32, fasted measurements of BMI and waist circumference were recorded. Height was measured to the nearest 0.1 cm using a wall-mounted stadiometer (screening visit only). BMI was calculated using the Tanita BC-418 digital scale (Tanita Europe), under normal settings (standard body type and −1 kg for clothing) with participants wearing light clothing. Waist circumference was measured by a trained investigator, in triplicate, halfway between the iliac crest and the lowest rib margin to the nearest 0.5 cm [31].

### Assessment of plasma phospholipid fatty acid status

Fasting blood samples were collected into lithium heparin tubes for determination of PL-FA status prior to and after each 12-week intervention period (week 0, 12, 20, 32). Chilled samples were centrifuged at 3000 rpm (1700 g) for 15 min at 4 °C and plasma stored at −80 °C until subsequent extraction and analysis.

Sequential multipurpose sampler systems (Gerstel GmbH & Co. KG, Mülheim an der Ruhr, Germany) were employed for automated sample preparation and derivatization of FAs from the phospholipid fraction at the MRC Human Nutrition Research, Cambridge, as previously described [43]. The phospholipid fraction was isolated from plasma using solid phase extraction on Na_2_SO_4_ 50 mg/NH_2_ 100 mg SPE cartridges (BE Gerstel; Agilent Technologies) and the FAME were produced from the phospholipid fraction according to the method published by Burge et al. [44]. The FAMEs were separated using a 30 m capillary column (HP-88; Agilent Technologies, Cheshire, UK) and detected using flame ionization. Results were expressed as molar percentage of total PL-FAs (mol%).

### Power calculation and statistical analyses

A total number of 54 participants were required for the chronic study to have sufficient power to detect a significant change in the primary and key secondary outcome measures. The primary outcome (serum total cholesterol, composed of LDL and HDL-cholesterol) was predicted to result in a ~0.3 mmol/L reduction with a population mean of 4.54 ± 0.5 mmol/L, with a power of 80% at *P* < 0.05, allowing for a 15% dropout rate. The chronic study was also powered to detect a 1.5% inter-group difference in the key secondary outcome measure, endothelial-dependent flow-mediated dilatation, with a power of 80% at *p* < 0.05 [45]. However, this paper is reporting on the food-exchange model and compliance to our high-fat, high-dairy interventions, which varied in FA composition.

Weighed dietary records, anthropometrics and PL-FA were analyzed using mixed models. Change-from-baseline for each variable of interest were modelled. Fixed effects included in the model were baseline values of the assessed variable, period, treatment, age, gender and BMI. All data were checked for normality and log transformed, if necessary. Participants were included as a fixed effect. There were no effects of the period in the model for any outcome measure.

Paired *t*-tests were used to assess differences in mean dietary compliance scores, as assessed by tick-sheet records, and underestimation of EI between the control and modified dietary interventions. For confirmation that our randomization approach was effective, differences in baseline characteristics between participants randomly assigned to the control and modified dietary intervention periods were assessed using independent *t*-tests and Chi-square tests for continuous and categorical variables, respectively. Statistical analyses were conducted using the SAS university edition statistical software (version 9.4; SAS Institute lnc., Cary, NC, USA). Results are presented as means and standard error of the mean (SEM). Differences were considered significant at *p* ≤ 0.01 to account for multiplicity.

Orthogonal partial least squares discriminant analysis (PLS-DA) was applied to identify patterns between variables in our PL-FA dataset and summarised it by reducing the number of dimensions or components [46]. Measured PL-FA concentrations of 36 individual FAs were subjected to orthogonal PLS-DA at: 1) baseline and 2) post-intervention using Metabolanalyst 3.0 [46]. The R^2^Y and Q^2^ values represented the goodness of fit and predictability of the models, respectively. Significance of the models were tested using 1000 permutations.

## Results

**Fig. 1:**
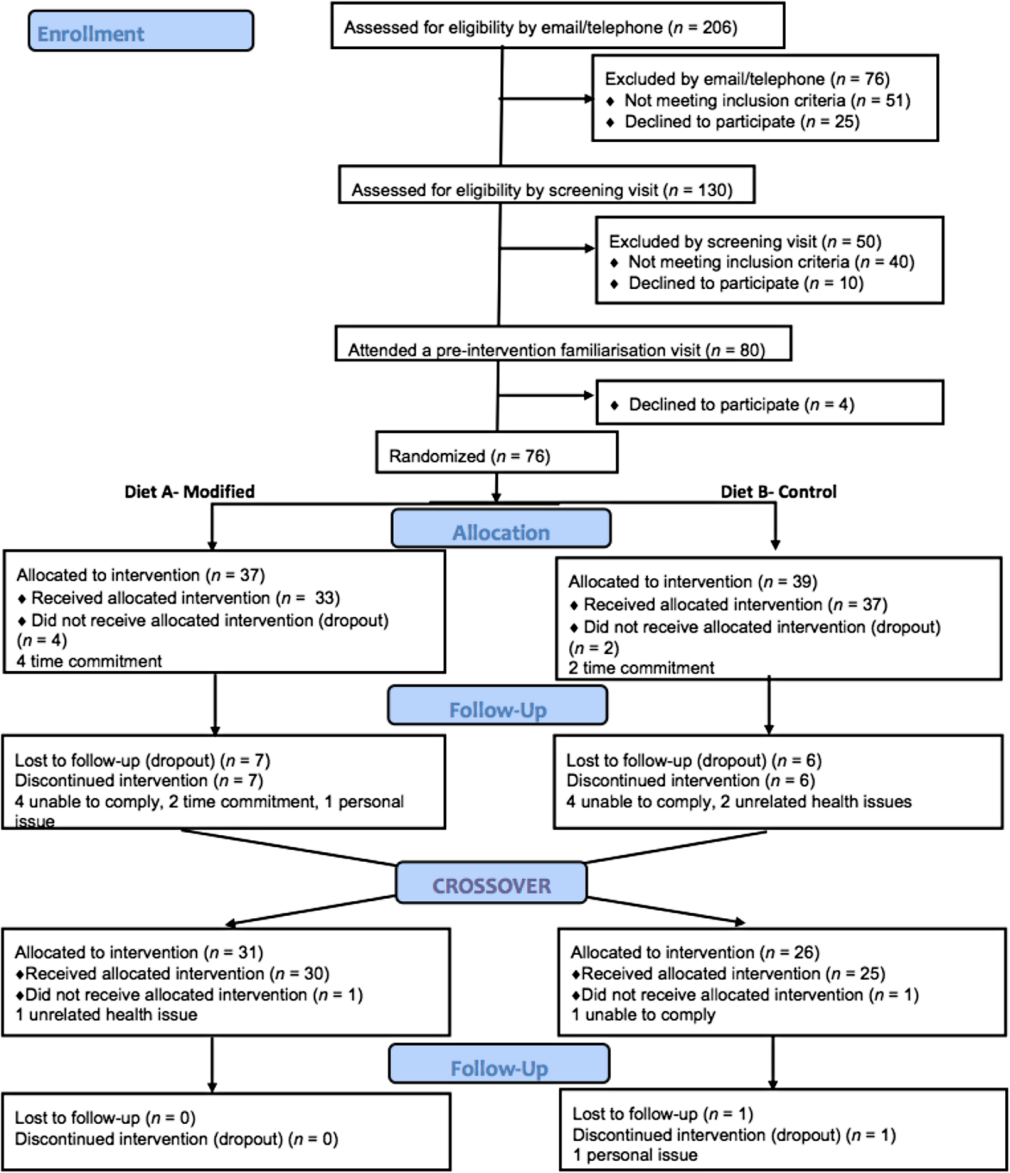
Flow of participants through the different stages of the RESET study

Participant flow through the study is illustrated in Fig. 1. Of the 74 participants who were randomly assigned and commenced the study, 54 (31 males and 23 females) completed the study. Participants continued to take their regular prescribed medication for the duration of the study (*n* = 2 levothyroxine for hypothyroidism; *n* = 1 proton pump inhibitor for heartburn; *n* = 1 hormone replacement therapy for menopausal symptom relief; *n* = 1 tricyclic antidepressant for depression; *n* = 2 fluticasone and salmeterol for mild asthma; *n* = 1 etonogestrel contraceptive implant and *n* = 1 levonorgestrel-releasing intrauterine contraceptive device). The breakdown of the ethnicity of the study group was as follows: White, 89% (*n* = 48); Asian, 4% (*n* = 2); Black, 4% (*n* = 2) and Chinese/Far Eastern, 4% (*n* = 2). There were no significant differences between participants randomly assigned to Groups 1 and 2 at baseline (Additional file 1: Table S1). Two participants were excluded from the dietary analysis due to insufficient data.

### Dietary analysis

Recorded dietary intake at baseline and following diets that incorporated the control and modified dairy products (post-intervention) are shown in Table 3. In agreement with target intakes (Table 2), significant treatment effects were evident for dietary intakes of SFA and MUFA. Following introduction of the modified diet, there was a smaller increase in SFA and greater increase in MUFA and TFA, relative to baseline, when compared with the control.

**Table 3:** Recorded dietary intake at baseline (week 0) and following diets that incorporated the control and modified dairy products (week 12) in adults at moderate cardiovascular disease risk and target fatty acid intakes^a^

	Control				Modified				
	Baseline	Post	T^b^	Δ	Baseline	Post	T	Δ	*p* ^c^
Energy, MJ/d	8.5 ± 0.4	9.0 ± 0.3		0.5 ± 0.3	8.2 ± 0.3	9.2 ± 0.4		1.0 ± 0.4	0.60
Total fat, %TE	36.5 ± 0.8	39.5 ± 0.7	38.0	3.0 ± 0.9	36.1 ± 0.8	41.1 ± 0.8	38.0	5.0 ± 1.1	0.03
SFA, %TE	13.9 ± 0.5	19.1 ± 0.4	19.0	5.2 ± 0.6	14.2 ± 0.5	16.9 ± 0.4	16.0	2.7 ± 0.6	<0.001
MUFA, %TE	11.9 ± 0.4	11.8 ± 0.3	11.0	−0.1 ± 0.3	11.7 ± 0.4	15.3 ± 0.4	14.0	3.6 ± 0.5	<0.0001
n-6 PUFA, %TE	4.6 ± 0.2	3.3 ± 0.2		−1.3 ± 0.2	3.9 ± 0.2	3.4 ± 0.2		−0.5 ± 0.2	0.08
n-3 PUFA, %TE	0.8 ± 0.1	0.6 ± 0.0		−0.2 ± 0.1	0.7 ± 0.0	0.6 ± 0.0		−0.1 ± 0.1	0.34
Total PUFA, %TE	5.8 ± 0.4	4.4 ± 0.5		−1.4 ± 0.3	4.6 ± 0.2	4.0 ± 0.2		−0.4 ± 0.2	0.14
TFA, %TE	1.0 ± 0.1	1.3 ± 0.1		0.3 ± 0.1	0.9 ± 0.1	2.5 ± 0.1		1.6 ± 0.1	<0.0001
Protein, %TE	16.3 ± 0.5	16.1 ± 0.4		−0.2 ± 0.5	16.9 ± 0.5	16.2 ± 0.3		−0.7 ± 0.6	0.80
Carbohydrates, %TE	46.3 ± 0.9	43.3 ± 0.9		−3.0 ± 0.9	46.6 ± 1.3	42.2 ± 1.0		−4.4 ± 1.5	0.19
Alcohol, %TE	3.1 ± 0.5	3.1 ± 0.5		0.0 ± 0.4	2.9 ± 0.5	2.7 ± 0.4		−0.2 ± 0.5	0.35
Dietary fiber (AOAC), g/d	20.4 ± 1.1	22.0 ± 1.1		1.6 ± 0.9	20.2 ± 1.1	19.4 ± 1.1		−0.8 ± 1.2	0.03
Sodium, g/d	2.7 ± 0.2	2.2 ± 0.1		−0.5 ± 0.1	2.5 ± 0.2	1.9 ± 0.1		−0.6 ± 0.1	0.03

Participant was included as a random effect. *p* ≤ 0.01 deemed as significant

*AOAC* Association of Official Analytic Chemists, *%TE* percentage of total energy

^a^Values are means ± SEM. Dietary intakes estimated from 4-d weighed dietary records at baseline (week 0) and after intervention (week 12)

^b^Target FA intakes for the control and modified dietary exchange periods

^c^Overall effect of treatment based on change-from-baseline was calculated by mixed model analysis, with adjustments made for fixed effects of baseline values of the assessed variable, period, treatment, age, gender and BMI

### Underestimation of energy intake

Based on the assumption that participants were in energy balance, it was estimated that recorded EI were underestimated for 35 and 22% of participants at baseline and post-intervention, respectively. Underestimation of EI was not considered further in the mixed model analysis.

### Dietary compliance

On the basis of tick-sheet records, mean daily intake (± SEM) of the dairy products across each 12-week dietary exchange was as follows: milk (control: 343.5 ± 1.8; modified: 347.8 ± 2.8 g/d), cheese (control: 46.5 ± 0.6; modified: 45.9 ± 0.4 g/d) and butter (control: 22.0 ± 0.2; modified: 25.6 ± 0.3 g/d). Mean daily dietary compliance did not vary according to treatment: milk (control: 96.6 ± 0.01; modified: 96.5 ± 0.01%; *p* = 0.92), cheese (control: 96.6 ± 0.01; modified: 96.8 ± 0.01%; *P* = 0.83) and butter (control: 96.5 ± 0.01; modified: 97.0 ± 0.01%; *p* = 0.70).

### Anthropometric measures

Relative to baseline, there was no significant treatment effect for BMI (control: 25.8 ± 0.5 vs. 26.2 ± 0.5 kg/m^2^ for baseline vs. post-intervention; modified: 25.8 ± 0.5 vs. 25.9 ± 0.5 kg/m^2^; *p* = 0.13) or waist circumference (control: 88.9 ± 1.4 vs. 89.8 ± 1.5 cm; modified: 89.3 ± 1.5 vs. 89.1 ± 1.5 cm; *p* = 0.53).

### Plasma phospholipid FA status

**Fig. 2:**
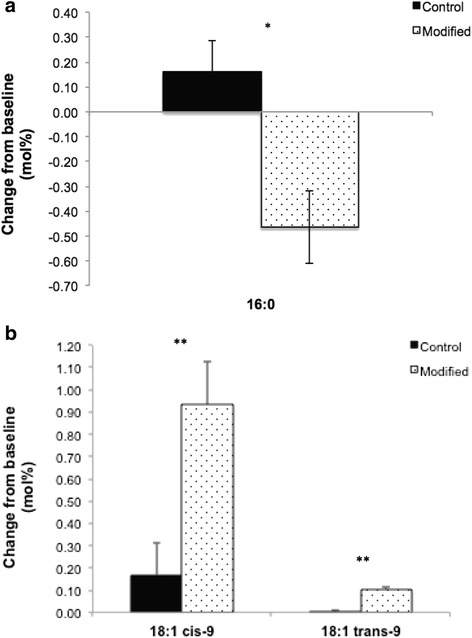
Change-from-baseline in the plasma phospholipid profile of SFA: 16 : 0 (**a**) and MUFAs: 18 : 1*cis*-9 and 18 : 1*trans*-9 (**b**) following 12-week diets that incorporated control and modified dairy products. Values are means ± SEM, *n* = 54. Significance shown as * *p* <0.001, ** *p* < 0.0001

After the 12-week intervention, there were significant differences in PL-FA composition between the modified and control groups (*p* ≤ 0.01). Consumption of the modified dairy products led to a small but significant decrease in abundance of total SFAs from baseline (change: −0.60 ± 0.21 mol%) vs. control (change: 0.01 ± 0.17 mol%) (Table 4). Following the modified diet, there were significant increases in *cis*-MUFAs and *trans*-MUFAs from baseline vs. control (Table 4). There was a minor increase in the abundance of 16:0 from baseline following the control diet (change: 0.16 ± 0.13 mol%), while a decrease from baseline was evident following the modified diet (change: −0.46 ± 0.15 mol%) (Fig. 2b). Compared with baseline, 18 : 1*cis*-9 and 18 : 1*trans*-9 increased following the modified diet vs. control (Fig. 2b). Following the control diet, there was a significant increase in 20 : 3*n*-6 vs. the modified diet.

**Table 4:** Plasma phospholipid fatty acids at baseline (week 0) and following diets that incorporated the control and modified dairy products (week 12) in adults at moderate cardiovascular disease risk^a^

	Control			Modified			
mol%	Baseline	Post	Δ	Baseline	Post	Δ	*p* ^b^
SFAs							
11:0	0.0000 ± 0.0008	0.0013 ± 0.0008	0.0000 ± 0.0011	0.0000 ± 0.0011	0.0002 ± 0.0000	0.0025 ± 0.0010	0.35
12:0	0.0230 ± 0.0018	0.0242 ± 0.0015	0.0012 ± 0.0019	0.0245 ± 0.0000	0.0225 ± 0.0000	0.0019 ± 0.0016	0.22
13:0	0.00 ± 0.00	0.00 ± 0.00	0.00 ± 0.00	0.00 ± 0.00	0.00 ± 0.00	0.00 ± 0.00	0.16
14:0	0.35 ± 0.01	0.42 ± 0.02	0.07 ± 0.02	0.35 ± 0.02	0.38 ± 0.02	0.03 ± 0.01	0.04
15:0	0.23 ± 0.01	0.26 ± 0.01	0.04 ± 0.01	0.22 ± 0.01	0.24 ± 0.01	0.02 ± 0.01	0.02
16:0	30.70 ± 0.16	30.86 ± 0.17	0.16 ± 0.13	30.78 ± 0.13	30.32 ± 0.13	−0.46 ± 0.15	<0.001
17:0	0.39 ± 0.01	0.40 ± 0.01	0.01 ± 0.01	0.38 ± 0.01	0.38 ± 0.01	0.00 ± 0.01	0.03
18:0	13.95 ± 0.18	13.67 ± 0.14	−0.29 ± 0.12	14.05 ± 0.12	13.83 ± 0.12	−0.22 ± 0.13	0.33
20:0	0.14 ± 0.00	0.13 ± 0.00	−0.01 ± 0.00	0.13 ± 0.00	0.12 ± 0.00	−0.01 ± 0.00	0.76
21:0	0.0128 ± 0.0037	0.0130 ± 0.0043	0.0001 ± 0.0036	0.0093 ± 0.0000	0.0147 ± 0.0000	0.0054 ± 0.0034	0.62
22:0	0.23 ± 0.01	0.22 ± 0.01	−0.01 ± 0.01	0.22 ± 0.01	0.22 ± 0.01	0.00 ± 0.01	0.69
23:0	0.10 ± 0.00	0.11 ± 0.00	0.01 ± 0.00	0.10 ± 0.00	0.11 ± 0.00	0.01 ± 0.00	0.34
24:0	0.25 ± 0.01	0.25 ± 0.01	0.00 ± 0.01	0.25 ± 0.01	0.24 ± 0.01	−0.01 ± 0.01	0.85
Total SFA^c^	46.37 ± 0.17	46.36 ± 0.13	0.01 ± 0.17	46.52 ± 0.21	45.92 ± 0.15	−0.60 ± 0.21	0.006
MUFAs							
18:1 *cis*-9	9.81 ± 0.15	9.98 ± 0.16	0.17 ± 0.15	9.99 ± 0.15	10.93 ± 0.15	0.93 ± 0.19	<0.0001
18:1 *trans*-9	0.08 ± 0.00	0.08 ± 0.00	0.01 ± 0.00	0.07 ± 0.00	0.17 ± 0.00	0.10 ± 0.01	<0.0001
22:1 *cis-9*	0.0202 ± 0.0037	0.0206 ± 0.0030	0.0004 ± 0.0045	0.0182 ± 0.0000	0.0201 ± 0.0000	0.0000 ± 0.0019	0.52
Total *cis*-MUFA^d^	11.02 ± 0.16	11.24 ± 0.17	0.21 ± 0.15	11.21 ± 0.18	12.20 ± 0.23	0.99 ± 0.20	<0.0001
Total *trans*-MUFA^e^	0.12 ± 0.00	0.12 ± 0.00	0.01 ± 0.00	0.10 ± 0.00	0.23 ± 0.01	0.12 ± 0.01	<0.0001
PUFAs							
18:2 n-6	22.26 ± 0.42	22.40 ± 0.37	0.14 ± 0.28	22.00 ± 0.28	22.29 ± 0.28	0.30 ± 0.29	0.87
18:3 n-6	0.08 ± 0.01	0.10 ± 0.01	0.02 ± 0.01	0.09 ± 0.01	0.09 ± 0.01	0.00 ± 0.01	0.51
18:3 n-3	0.31 ± 0.02	0.31 ± 0.01	0.00 ± 0.01	0.31 ± 0.01	0.29 ± 0.01	−0.02 ± 0.01	0.58
20:3 n-6	3.07 ± 0.08	3.39 ± 0.11	0.32 ± 0.07	3.12 ± 0.07	3.17 ± 0.07	0.06 ± 0.06	0.007
20:4 n-6	9.88 ± 0.27	9.53 ± 0.22	−0.35 ± 0.13	9.77 ± 0.13	9.41 ± 0.13	−0.36 ± 0.21	0.74
20:5 n-3	1.27 ± 0.08	1.25 ± 0.06	−0.02 ± 0.07	1.24 ± 0.07	1.14 ± 0.07	−0.10 ± 0.07	0.14
22:5 n-6	0.20 ± 0.01	0.20 ± 0.01	0.00 ± 0.01	0.20 ± 0.01	0.20 ± 0.01	0.00 ± 0.01	0.17
22:5 n-3	0.99 ± 0.03	1.02 ± 0.02	0.03 ± 0.02	1.00 ± 0.02	0.95 ± 0.02	−0.05 ± 0.03	0.55
22:6 n-3	3.77 ± 0.16	3.39 ± 0.13	−0.37 ± 0.09	3.79 ± 0.09	3.44 ± 0.09	−0.35 ± 0.10	0.74
Total n-3 PUFA^f^	5.35 ± 0.23	4.96 ± 0.17	−0.39 ± 0.14	5.34 ± 0.21	4.87 ± 0.17	−0.47 ± 0.14	0.58
Total n-6 PUFA^g^	36.15 ± 0.34	36.30 ± 0.27	0.15 ± 0.26	35.83 ± 0.32	35.86 ± 0.33	0.03 ± 0.33	0.32

Where no bond position is listed it is unknown [40]. 20 : 4*n*-6 + 20 : 3*n*-3 co-eluted, but as 20 : 3*n*-3 concentration in human samples is negligible, this peak was identified as 20 : 4*n*-6 [43]

^a^Values are given as means ± SEM

^b^Overall effect of treatment based on change-from-baseline was calculated by mixed model analysis, with adjustments made for fixed effects of baseline values of the assessed variable, period, treatment, age, gender and BMI. Participant was included as a random effect

^c^Total SFAs include: 11 : 0, 12 : 0, 13 : 0, 14 : 0, 15 : 0, 16 : 0, 17 : 0, 18 : 0, 20 : 0, 21 : 0, 22 : 0, 23 : 0 and 24 : 0

^d^Total *cis*-MUFAs include: 14 : 1*cis*, 15 : 1*cis*, 16 : 1*cis*, 17 : 1*cis*, 18 : 1*cis*-9, 20 : 1*cis*, 22 : 1*cis*-9 and 24 : 1*cis*. Where no bond position is listed it is unknown, as previously outlined in Wang et al. [43]

^e^Total *tran*s-MUFAs include: 16 : 1*trans* (bond position unknown) and 18 : 1*trans*-9 [43]

^f^Total *n*-3 PUFAs include: 18 : 3*n*-3, 20 : 5*n*-3, 22 : 5*n*-3 and 22 : 6*n*-3

^g^Total *n*-6 PUFAs include: 18 : 2*n*-6, 18 : 2*trans*, 18 : 3*n*-6, 20 : 2, 20 : 3*n*-6, 20 : 4*n*-6 + 20:3*n*-3, 22 : 4 and 22 : 5*n*-6

### Orthogonal Partial Least Square Discriminant analysis of plasma phospholipid FA data

**Fig. 3:**
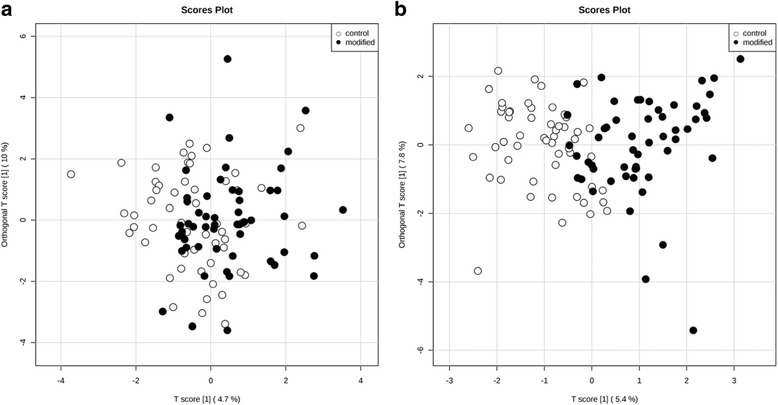
Orthogonal PLS-DA, score plots at baseline (**a**) and post-intervention (**b**) calculated using plasma phospholipid FA concentrations in adults at moderate risk of cardiovascular disease (*n* = 54). FA, fatty acid; PLS-DA, partial least squares discriminant analysis

For the baseline PLS-DA, the first component representing the maximum differentiation between the two diets represented 4.7% of variation and was retained to interpret the FA profiles of the clusters on the score plots. PLS-DA of the PL-FA data revealed a lack of distinction between the control and modified intervention groups at baseline (R^2^Y = 0.142 and Q^2^ = −0.35, empirical *P*-values R^2^Y: *p* = 0.99 (986/1000) and Q^2^: *P* = 0.76 (763/1000), suggesting that the population was indistinguishable with regard to PL-FA profiles prior to commencement of the dietary exchange periods (Fig. 3a). Additional file 2: Table S2 illustrates the loadings.

Following the intervention, the first component representing the maximum differentiation between the two diets represented 5.4% of the variability in the data. The loadings are illustrated in Additional file 2: Table S2. In contrast to baseline, the post-intervention score plot identified a clear separation (R^2^Y = 0.612 and Q^2^ = 0.451, empirical *p*-values R^2^Y: *p* < 0.001 (0/1000) and Q^2^: *p* < 0.001 (0/1000) in PL-FA profiles of participants when they were assigned to the modified and control intervention diets (Fig. 3b). The FAs that mainly contributed to this dietary status separation were: 18 : 1*trans-9*, 16 : 1*trans*, 18 : 1*cis*-9, that were higher following the modified diet, while 16 : 0, 14 : 1 *cis*, 14 : 0, 15 : 0 and 20 : 3*n*-6 were higher following the control diet.

## Discussion

The food-exchange model was used for the implementation of two iso-energetic high-fat, high-dairy diets varying in FA composition in the RESET study, through the use of SFA-reduced, MUFA-enriched dairy products and control alternatives with a FA profile typical of retail dairy products. Specific dietary targets following treatments were largely achieved in a free-living population at moderate risk of CVD. Analysis of weighed dietary records confirmed that it was possible to lower the mean SFA intake by 2.5%TE and increase MUFA intake by 3.7%TE following the diet containing modified dairy products compared with the control products. Previously, Noakes et al. reported differences of 2.2%TE SFA and 2.8%TE MUFA (described as oleic acid intake only) following consumption of dairy products that were produced following a protein-encapsulated lipid (rapeseed and soybean oil) bovine supplementation period, when compared to control dairy foods [47]. There was a significant difference in recorded SFA intake between interventions in our study, however the reduction in SFA intake following the modified diet was slightly less than predicted by our food-exchange model. Compliance was further confirmed by assessment of dietary tick-sheet records.

To our knowledge, this is the first human study to assess the impact of modified milk, cheese and butter consumption on PL-FA concentrations, in comparison to control dairy products with a FA profile typical of retail products. In line with previous findings [31, 48–50], objective dietary compliance was confirmed by assessment of PL-FA profiles, with consumption of the control and modified dairy products leading to differential effects on total plasma phospholipid SFAs and MUFAs and their sub-classes. The consumption of the SFA-reduced, MUFA-enriched dairy products led to a small decrease in the abundance of total SFAs, and increases in total *cis*-MUFAs, including 18 : 1*cis*-9 in the PL-FA profile, when compared to the control products. These changes are comparable to the proportion of PL-FA total SFAs and MUFAs observed in the DIVAS study following a 16-week MUFA-rich diet (9%TE SFA; 19%TE MUFA; 4%TE *n*-6 PUFA) [31]. Previous literature has suggested that SFAs and MUFAs with even numbered carbon chain length can be endogenously synthesised by humans and may be less affected by dietary intake [51]. Hodson et al. suggested that this may be because it is difficult to alter the proportion of FAs that are already relatively abundant in the diet and that increases in SFA intake are not reflected in increases in the plasma FA profiles [30]. However, changes in PL-FA concentrations observed in the RESET study mirrored the FA composition of our intervention dairy foods [52]. PLS-DA provided a means for visualizing adherence to a dietary intervention. Despite using a relatively homogenous population and a modest dietary exchange, our PLS-DA plot highlighted some distinction between the PL-FA profile of the participants following the modified and control dietary exchange periods, suggesting a small but significant response to our intervention. This analysis only explained approximately 5% of the overall variability in our dataset but the affected FAs, including 18 : 1*trans*-9 and 18 : 1*cis*-9 and 16 : 0, were relevant to our interevntions. Furthermore, it provided further evidence that PL-FA were illustrative of short to medium-term FA intake [29–31].

Supplementation of the dairy cow diet with unsaturated FA leads to increased levels of ruminant *trans*-fatty acids (rTFA) in the milk and dairy products, through ruminal biohydrogenation of unsaturated FA [15, 25, 27]. There was a calculated dietary increase of 1.3%TE in total TFA intake following the modified, when compared with the control diet. It was not possible to quantify our participants’ voluntary intake of specific TFA isomers (i.e. ruminant or industrial TFA intake) from weighed dietary records using our nutrient analysis software. However, based on the differences in TFA composition between our control and modified dairy products [52], it is apparent that the majority of the increased TFA intake recorded following the modified diet was derived from ruminant sources. In contrast to the recognised detrimental impact of industrially-produced TFA on cardiovascular health, consumption of rTFA may not be adversely linked to CVD risk [53], except possibly at high intakes [54, 55]. It should be noted that the calculated dietary TFA intake following our modified intervention (2.5 ± 0.1%TE) exceeded the recommended population maximum of 2% food energy [3] which does not discriminate between ruminant and industrial sources, and is higher than the current mean TFA intake in UK adults (0.5%TE and 0.5% food energy) [4]. This may be in part explained by the high dairy fat content of our intervention diets. In support of this we also observed that consumption of the SFA-reduced, MUFA-enriched dairy products lead to significant increases in total *trans*-MUFA and 18:1 *trans-9* concentrations in the PL-FA. Whilst our PL-FA analysis approach was unable to identify specific *trans* isomers other than 18:1 *trans-9* [43], the detailed FA analysis of the dairy foods used in the RESET study (Kliem KE, Humphries DJ, Markey O, Vasilopoulou D, Fagan CC, Grandison AS, Todd S, Givens DI, Lovegrove JA: Food chain approach to lowering saturated fat in milk and dairy products: the RESET study, submitted) suggest there was likely to be a complex mixture of TFA in the phospholipid pool. It may be that feeding strategies that limit increases of TFA in milk fat following bovine supplementation with plant oils, such as the use of encapsulation protection technology may be advantageous [27, 56]. However, further work is justified to determine whether rTFA are detrimental to cardiovascular health [27].

Our intervention presented some challenges. Our bovine supplementation strategy was successful in altering the FA profile of the milk [52]. Alongside this, we observed a depression in milk fat content following supplementation of the bovine diet with high-oleic sunflower oil which has been reported previously [57]. As a result of this, it was necessary to standardise the fat content of the raw control milk prior to UHT so that it was equivalent to that of the modified milk. Furthermore, our modified cheese had a lower fat content compared with conventional cheese and it was necessary for our participants to consume an additional 3.6 g/d of butter during the modified dietary exchange period to standardize the fat intake of the two intervention periods.

A challenge faced by some participants was incorporating sufficient quantities of products, especially cheese, into their habitual diets on a daily basis. In line with recent NDNS data [4], some of our participants were not accustomed to consuming the quantities of dairy products that were prescribed in our food-exchange model (unpublished data obtained by food frequency questionnaire). Although it was not logistically feasible for us to include a wider range of dairy products in the RESET study, it is possible that a greater variety of items may have reduced the likelihood of ‘product boredom’ and minimized the potential for compliance issues [58]. Compliance with the dietary exchange was cited as the main reason for dropout by nine of our participants, predominantly in the early stages of the intervention. It should be noted that a similar number of participants withdrew from the modified and control dietary exchange periods, suggesting that the two regimens were equal in terms of acceptability. For those who completed the study, our tick-sheet records suggested that compliance to the dietary regimens was excellent and our PL-FA data provided further objective evidence of dietary adherence. It is acknowledged that there may have been discrepancies in tick-sheet recordings as a result of ‘desirability bias’, i.e. participants may have recorded the portions that they were required to eat as opposed to what they had actually consumed [58], and this may have led to more modest differences in PL-FA profiles between the two dietary exchange periods.

We observed a moderately high amount of under-reporting assessed by EI at baseline, however, this was similar to that observed in previous free-living populations [31, 59]. A high proportion of participants (61%; *n* = 33) in the RESET study were classified as overweight (BMI of ≥ 25.0 kg/m^2^). It is recognized that overweight or obese individuals may be more prone to selective bias and omission of foods with a negative health image, leading to potential under-reporting of dietary intake [60]. We observed a lower degree of under-reporting following the intervention, when our replacement model predicted that the dairy products would contribute to over 25% of daily EI. It could be speculated that participants had a greater appreciation of the importance of giving an accurate account of their dietary intake whilst on the intervention. The present study has illustrated that through the incorporation of dairy products with an altered FA profile into the habitual diet, it was possible for free-living participants to successfully improve their dietary fat quality. This was in agreement with previous successful food-exchange models [31, 35, 58]. Our dietary interventions were designed to contain a high quantity of dairy fat (~41 g/d). Both intervention periods increased relative intake of total fat and SFA over baseline. However, it is feasible that a similar, but more moderate, approach could be used for assisting in the reduction of dairy SFA intake at a population level, without reducing dairy consumption. Furthermore, we have previously reported that consumers generally accepted the SFA-reduced, MUFA-enriched dairy products, when tasted in a blinded manner (Markey O, Souroullas K, Fagan CC, Kliem KE, Vasilopoulou D, Jackson KG, Humphries DJ, Grandison AS, Givens DI, Lovegrove JA, Methven L: Consumer acceptance of dairy products with a saturated fatty acid-reduced, monounsaturated fatty acid-enriched content, In Review).

## Conclusions

We sucessfully implemented a high-fat, high-dairy food-exchange model that was suitable for replacing dairy products with a FA profile typical of retail products with SFA-reduced, MUFA-enriched alternatives over a 12-week period in a free-living population at moderate risk of CVD. Changes in the dietary intake of SFA and MUFA between our interventions was confirmed by changes in the concentrations of these FAs in the plasma phospholipid fraction, indicative of adherence to our dietary intervention.

## Additional files

Additional file 1: Table S1. Baseline characteristics for participants’ based on the order of allocation to the control and modified dietary exchange periods. (DOC 36 kb)

Additional file 2: Table S2. Factor loadings identified by orthogonal partial least squares discriminant analysis of plasma phospholipid fatty acid profiles at baseline (week 0) and following diets that incorporated the control and modified dairy products (week 12) in adults at moderate cardiovascular disease risk. (DOC 73 kb)

## Abbreviations

AOAC: Association of Official Analytic Chemists
%TE: Percentage of total energy
Ca: Calcium
CVD: Cardiovascular disease
DIVAS: Dietary Intervention and Vascular Function
FA: Fatty acid
Mg: Magnesium
Na: Sodium
NDNS: National Diet and Nutrition Survey
P: Phosphorus
PC: Principal component
PL-FA: Plasma phospholipid FA
PLS-DA: Partial least squares discriminant analysis
RCT: Randomized controlled trial
RISCK: Reading, Imperial, Surrey, Cambridge, and Kings
rTFA: Ruminant *trans*-fatty acids
TFA: *Trans*-fatty acid
UHT: Ultra high temperature
