# Supplementary material for: Plasma phospholipid fatty acid profile confirms compliance to a novel saturated fat-reduced, monounsaturated fat-enriched dairy product intervention in adults at moderate cardiovascular risk: a randomized controlled trial
Source: Nutr J. 2017 May 23;16:33. doi: 10.1186/s12937-017-0249-2 (PMC5442645; doi:10.1186/s12937-017-0249-2)
Supplement: Supplementary file 1 — Baseline characteristics for participants’ based on the order of allocation to the control and modified dietary exchange periods. (DOC 36 kb) [file 12937_2017_249_MOESM1_ESM.doc]

**Additional file 1: Table S1** Baseline characteristics for participants’ based on the order of allocation the control and modified dietary exchange periods^a^

|  | Group 1^b^ | Group 2 | *P*^c^ |
| --- | --- | --- | --- |
|  | (*n* = 30) | (*n* = 24) |  |
| Age, *y* | 52 ± 2 | 51 ± 3 | 0.41 |
| Gender, *%M/%F* | 53/47 | 63/38 | 0.50 |
| BMI, *kg/m* | 25.0 ± 0.6 | 25.8 ± 0.7 | 0.99 |
| Waist circumference, *cm* | 86.7 ± 1.9 | 90.2 ± 2.2 | 0.83 |
| Fasting total cholesterol, *mmol/L* | 5.60 ± 0.20 | 5.45 ± 0.18 | 0.80 |
| Fasting HDL-cholesterol, *mmol/L* | 1.46 ± 0.04 | 1.49 ± 0.08 | 0.92 |
| Fasting glucose, *mmol/L* | 5.37 ± 0.17 | 5.46 ± 0.22 | 0.84 |
| Systolic blood pressure, *mmHg*^d^ | 119 ± 2 | 122 ± 2 | 0.67 |
| Diastolic blood pressure, *mmHg*^d^ | 71 ± 1 | 74 ± 2 | 0.82 |
| Family history of premature MI, *%*^e^ | 16.7 | 12.5 | 0.36 |
| Risk Score^f^ | 3.2 ± 0.3 | 2.8 ± 0.2 | 0.28 |

^a^All values are given as mean ± SEM; ^b^Group 1= participants were randomly allocated to consume control dairy products during their first dietary exchange period. Group 2= participants were randomly allocated to consume modified (SFA-reduced, MUFA-enriched) dairy products during their first dietary exchange period; ^c^Differences in baseline characteristics between participants randomly assigned to the control and modified dietary exchange periods were assessed using independent *t*-tests and Chi-square tests for continuous and categorical variables, respectively. *P* ≤ 0.01 deemed as significant; ^d^Measured over 24 h using ambulatory blood pressure monitors approximately 48 h before clinical visit 1 at wk 0; ^e^Family history of prematurely diagnosed MI in parents or siblings (≤55 years for male relatives; ≤65 years for female relatives); ^f^A score of ≥2 points relates to a 50% greater risk of CVD than the population mean.
