# Supplementary material for: Plasma phospholipid fatty acid profile confirms compliance to a novel saturated fat-reduced, monounsaturated fat-enriched dairy product intervention in adults at moderate cardiovascular risk: a randomized controlled trial
Source: Nutr J. 2017 May 23;16:33. doi: 10.1186/s12937-017-0249-2 (PMC5442645; doi:10.1186/s12937-017-0249-2)
Supplement: Supplementary file 2 — Factor loadings identified by orthogonal partial least squares discriminant analysis of plasma phospholipid fatty acid profiles at baseline (week 0) and following diets that incorporated the control and modified dairy products (week 12) in adults at moderate cardiovascular disease risk. (DOC 73 kb) [file 12937_2017_249_MOESM2_ESM.doc]

**Additional file 2: Table S2** Factor loadings identified by orthogonal partial least squares discriminant analysis of plasma phospholipid fatty acid profiles at baseline (week 0) and following diets that incorporated the control and modified dairy products (week 12) in adults at moderate cardiovascular disease risk

|  | Baseline | |  | Post-intervention | |
| --- | --- | --- | --- | --- | --- |
|  | Loading (t1) | OrthoLoading (to1) |  | Loading (t1) | OrthoLoading (to1) |
| 10:0 | -0.008 | 0.153 |  | -0.038 | 0.109 |
| 11:0 | 0.167 | -0.076 |  | -0.116 | -0.193 |
| 12:0 | 0.013 | 0.158 |  | -0.095 | -0.055 |
| 13:0 | 0.089 | 0.111 |  | -0.011 | -0.312 |
| 14:0 | -0.027 | 0.272 |  | -0.238 | 0.224 |
| 14:1 *cis* | 0.247 | 0.000 |  | -0.255 | 0.005 |
| 15:0 | -0.291 | -0.107 |  | -0.226 | 0.188 |
| 15:1 *cis* | 0.292 | -0.031 |  | 0.103 | -0.285 |
| 16:0 | 0.105 | 0.129 |  | -0.247 | -0.085 |
| 16:1 *cis* | -0.451 | -0.093 |  | 0.392 | 0.264 |
| 16:1 *trans* | 0.036 | 0.233 |  | -0.001 | 0.182 |
| 17:0 | -0.216 | -0.365 |  | -0.147 | 0.115 |
| 17:1 *cis* | 0.221 | 0.019 |  | -0.123 | 0.174 |
| 18:0 | 0.222 | 0.026 |  | 0.088 | 0.033 |
| 18:1 *cis*-9 | -0.383 | 0.053 |  | 0.579 | 0.235 |
| 18:1 *trans*-9 | 0.061 | 0.277 |  | 0.258 | 0.374 |
| 18:2 *cis*-6 | -0.177 | -0.152 |  | 0.070 | 0.038 |
| 18:2 *trans*-6 | -0.148 | -0.065 |  | 0.023 | -0.101 |
| 18:3 n-6 | 0.118 | 0.241 |  | -0.136 | 0.115 |
| 20:0 | -0.181 | -0.262 |  | -0.053 | -0.357 |
| 18:3 n-3 | -0.038 | 0.182 |  | -0.152 | 0.102 |
| 20:1 *cis* | -0.403 | 0.126 |  | 0.186 | 0.443 |
| 21:0 | -0.094 | -0.214 |  | 0.068 | -0.123 |
| 20:2 | -0.129 | -0.026 |  | -0.037 | 0.030 |
| 20:3 n-6 | 0.014 | 0.221 |  | -0.158 | 0.214 |
| 22:0 | -0.095 | -0.423 |  | 0.087 | -0.297 |
| 20:4 n-6 | -0.037 | -0.056 |  | 0.009 | -0.132 |
| 22:1 *cis*-9 | -0.078 | 0.023 |  | -0.042 | -0.003 |
| 23:0 | -0.084 | -0.427 |  | -0.056 | -0.240 |
| 20:5 n-3 | -0.042 | -0.094 |  | -0.146 | 0.065 |
| 24:0 | 0.052 | -0.220 |  | 0.040 | -0.255 |
| 22:4 | 0.044 | 0.083 |  | -0.062 | -0.046 |
| 24:1 *cis* | -0.113 | -0.432 |  | -0.021 | -0.495 |
| 22:5 n-6 | 0.063 | 0.052 |  | 0.023 | 0.070 |
| 22:5 n-3 | 0.105 | -0.072 |  | -0.201 | 0.045 |
| 22:6 n-3 | 0.065 | -0.261 |  | 0.020 | -0.217 |
